# Knowledge, attitudes and practices towards pandemic influenza among cases, close contacts, and healthcare workers in tropical Singapore: a cross-sectional survey

**DOI:** 10.1186/1471-2458-10-442

**Published:** 2010-07-28

**Authors:** Jonathan Yap, Vernon J Lee, Teng Yan Yau, Tze Pin Ng, Phern-Chern Tor

**Affiliations:** 1 Biodefence Centre, Ministry of Defence, Singapore; 2 Department of Epidemiology and Public Health, National University of Singapore, Singapore; 3 Centre for Health Services Research, National University of Singapore, Singapore; 4 National Centre for Epidemiology and Population Health, Australian National University, Australia; 5 Healthcare Branch, Ministry of Defence, Singapore; 6 Department of Psychological Medicine, National University Hospital, Singapore

## Abstract

**Background:**

Effective influenza pandemic management requires understanding of the factors influencing behavioral changes. We aim to determine the differences in knowledge, attitudes and practices in various different cohorts and explore the pertinent factors that influenced behavior in tropical Singapore.

**Methods:**

We performed a cross-sectional knowledge, attitudes and practices survey in the Singapore military from mid-August to early-October 2009, among 3054 personnel in four exposure groups - laboratory-confirmed H1N1-2009 cases, close contacts of cases, healthcare workers, and general personnel.

**Results:**

1063 (34.8%) participants responded. The mean age was 21.4 (SE 0.2) years old. Close contacts had the highest knowledge score (71.7%, p = 0.004) while cases had the highest practice scores (58.8%, p < 0.001). There was a strong correlation between knowledge and practice scores (r = 0.27, p < 0.01) and knowledge and attitudes scores (r = 0.21, p < 0.01). The significant predictors of higher practice scores were higher knowledge scores (p < 0.001), Malay ethnicity (p < 0.001), exposure group (p < 0.05) and lower education level (p < 0.05). The significant predictors for higher attitudes scores were Malay ethnicity (p = 0.014) and higher knowledge scores (p < 0.001). The significant predictor for higher knowledge score was being a contact (p = 0.007).

**Conclusion:**

Knowledge is a significant influence on attitudes and practices in a pandemic, and personal experience influences practice behaviors. Efforts should be targeted at educating the general population to improve practices in the current pandemic, as well as for future epidemics.

## Background

In April 2009, a novel strain of Influenza A (H1N1) surfaced and has since spread widely across the globe with substantial clinical impact [1]. Effective pandemic management requires support from the population at risk for measures undertaken to mitigate the pandemic's spread. Previous studies during the Severe Acute Respiratory Syndrome (SARS) outbreak in 2003 have shown that individual beliefs and perceptions play an important role in subsequent desired behavior change [2,3]. Higher perceived effectiveness of measures undertaken [3,4] and higher perceived threat of the disease led to higher rates of positive behavioral change, and better knowledge also increased the uptake of preventive measures [5,6]. Similarly, in an anticipated H5N1 epidemic these factors also influenced both self and community protective behavior [7].

During the current influenza pandemic, studies have found that the individual's emotional status mediates behavioral response [8] and that perceived severity and susceptibility to disease and perceived effectiveness of specific behaviors resulted in the corresponding recommended behavior changes [9,10]. To increase positive perceptions, clear dissemination of information served to reduce misconceptions [11]. At the same time, interactions with family and friends influenced behavior, and distinct regional differences in behavioral responses were noted across various countries which may be due to socio-cultural differences [12].

It is therefore important to perform behavioral studies in different populations to understand the determinants that influence behaviors. In tropical regions, influenza exhibits different seasonal patterns with a high baseline influenza-like illness rates and multiple influenza epidemic peaks annually [13]. This may result in different behaviors towards influenza compared to temperate countries with clear influenza seasons during winter months - for example the generally lower influenza vaccination uptake in the tropics [14]. However, there have been few studies on the knowledge, attitudes and practices towards the influenza pandemic in a tropical setting. There have also been no studies comparing such differences in various cohorts such as influenza cases, close contacts, and healthcare workers. As such, there is a need to understand the factors influencing such behavioral changes to promote effective management of influenza pandemics in the tropical setting.

Singapore, a tropical island city-state in South-East Asia, experienced the local spread of pandemic influenza A (H1N1-2009) from June to October in a single typical epidemic wave. Our study, conducted in the Singapore military, aims to study the differences in knowledge, attitudes and practices in various different cohorts and explore the pertinent factors that influenced behavior.

## Methods

We performed a cross-sectional survey in the Singapore military from mid-August 2009 to early-October 2009, after the peak of the local epidemic which occurred during the first week of August in Singapore [15]. The Singapore military comprises of conscript males who serve compulsory military service after high school, and non-conscript regular servicemen. Most servicemen stay in camp during weekdays and return to the community/home during weekends, resulting in interactions within the military and general communities.

As part of the military's pandemic response plan, every serviceman was given an information pamphlet on pandemic influenza, with information about the virus and preventive measures that could be taken to reduce risk of transmission and infection. The servicemen were also briefed on the above with emphasis on personal hygiene measures and socially responsible behavior (for example covering the nose and mouth when sneezing and coughing). Other measures implemented include daily temperature monitoring, prompt reporting of all illnesses to healthcare staff, and laboratory testing of influenza-like illness clusters. Laboratory confirmed cases of H1N1-2009 were isolated at home for 7 days, while close contacts of these confirmed cases were allowed to continue working but were given post-exposure chemoprophylaxis with oseltamivir and were segregated from the rest of the military units to prevent spread. In addition, all healthcare workers were required to don personal protective equipment including N-95 masks during their working hours.

Our study population consisted of 4 distinct exposure groups of military personnel - laboratory confirmed H1N1-2009 cases, close contacts of H1N1-2009 cases (defined as those who had worked or lived with a laboratory confirmed H1N1-2009 case during the infectious period), healthcare workers, and other general servicemen. The military maintained a comprehensive list of all laboratory confirmed cases of H1N1-2009 and their close contacts, which were identified via contact tracing. An anonymous self-administered paper questionnaire was mailed to the respective servicemen with a self addressed envelope included for return of the survey forms. Questionnaires were sent to all laboratory confirmed cases and their contacts, all healthcare workers in the military and general servicemen from various representative units in the military. Approval for the study was obtained from the military's research committee.

### Questionnaire

We developed a questionnaire to assess the knowledge, attitudes and practices (KAP) regarding pandemic influenza. The questionnaire was based on similar questionnaires on this topic [9,16], as well as concepts of health behaviours [17,18], and was pilot tested among military servicemen with similar profiles to the actual cohort. The questionnaire collected basic demographic data on age, sex, ethnicity, education level and housing type while is a commonly-used national proxy for socio-economic status [19]; and included questions on knowledge, attitudes, and practices on pandemic influenza. Questions on knowledge were used to assess a servicemen's general knowledge on pandemic influenza and on the recommended response measures. Questions on attitudes were used to assess perceptions towards pandemic influenza and these measures. Questions on practices were used to assess the actual compliance and practice of these measures. A summary of the questions assessed are shown in Table 1. Servicemen were asked to rate their agreement with the statements in the questionnaire, which were scored either yes/no or on a 4 point Likert scale.

**Table 1:** Summary of Questions for Knowledge, Attitudes and Practices towards Pandemic Influenza A (H1N1)

Knowledge
**Basic Knowledge**

What is pandemic Influenza A (H1N1)?

Where did pandemic Influenza come from?

How does pandemic Influenza spread?

What are the symptoms of pandemic Influenza?

**Mask Knowledge**

N-95 masks are effective in reducing the spread of pandemic Influenza.

Surgical/Paper masks are effective in reducing the spread of pandemic Influenza.

**Vaccination Knowledge**

Influenza vaccination is an effective measure against influenza.

**Tamiflu Knowledge**

Tamiflu is effective for treatment of pandemic Influenza A (H1N1).

Tamiflu is effective for prophylaxis (prevention) against pandemic Influenza A (H1N1).

**Avoidance Behaviour Knowledge (The following will decrease my chance of catching pandemic influenza)**

Avoiding overseas travel to affected areas

Avoiding people with flu symptoms

Avoiding going outdoors

Avoiding crowded places

Avoiding public transport

Avoiding large social gatherings

**Personal Habits Knowledge (The following will reduce the spread of influenza)**

Washing my hands regularly with soap and water

Covering my mouth when I cough/sneeze

Daily temperature taking and symptoms monitoring

Staying away from others when I am ill

**Attitudes**

**Mask Attitudes**

I think it is good to wear masks at home.

I think it is good to wear masks in public places.

**Vaccination Attitudes**

I will be vaccinated against pandemic Influenza A (H1N1) if available.

Do you feel that the influenza vaccine has any side effects?

**Tamiflu Attitudes**

I would complete a course of Tamiflu if prescribed.

Do you feel that Tamiflu has any side effects?

**Medical Attention Seeking Attitudes**

I would visit a doctor if I am ill.

I would avoid hospital/GP clinic/medical centre to decreases my risk of catching pandemic influenza.

**Risk Perception Attitudes**

It is likely that I will catch the pandemic virus.

How long do you think the pandemic influenza infection will last?

Are you worried or distressed about the pandemic?

**Practice (over past 2 months)**

**Mask Practice**

How often have you worn masks at home?

How often have you worn masks in public areas?

**Vaccination Practice**

Have you had your seasonal influenza vaccination?

**Avoidance Behaviour Practice**

Have you avoided travelling to affected countries?

Have you avoided people with flu symptoms?

Have you avoided going outdoors?

Have you avoided crowded places?

Have you avoided public transport?

Have you avoided social gatherings?

**Personal Habits Practice**

Do you wash your hands regularly with soap and water ?

Do you cover your mouth when you cough/sneeze ?

Do you take your temperature and monitor for respiratory symptoms daily?

**Medical Attention Seeking Practice**

Have you avoided going to hospital/GP clinic/medical centre?

Did you seek medical consult if you had any flu-like symptoms?

### Statistical Analysis

To determine the sample size, we assumed that responses within each group were normally distributed with standard deviation of 5%. To detect a true difference in means between groups of 2%, we will need 132 participants per group to achieve power of 0.9 and p = 0.05. Due to the smaller number of known patients and contacts available, we mailed out more survey forms to general servicemen from various units to provide a larger control group and to account for non-response.

To determine the scores for each individual, the responses were recoded to 0 for an undesired response and 1 for a desired response with the exception of risk perception questions which were scored proportionate to the level of risk perception. The mean scores were summarized as percentages.

Chi-squared tests of significance were used for analyses of categorical variables, and analyses of variance were used for continuous variables among the four exposure groups of servicemen (patients, contacts, medical personnel and other servicemen). The relationships between knowledge, attitudes and practice scores were examined using bivariate correlational analyses and multivariate linear regression models. All the demographic variables were found to be significant predictors in the bivariate analyses in at least one exposure group of servicemen, and were hence included in the multivariate models, which included the practice scores and attitude scores as the dependent variables. All statistical analyses were performed using SPSS 14.0 for Windows (SPSS Inc, Chicago, IL), with the level of significance set at 5%.

## Results

A total of 3054 survey forms were mailed out (465 patients/contacts, 1568 healthcare workers and 1021 to general servicemen). The overall response rate was 34.8% (1063/3054). The response rates for patients/contacts, healthcare workers and other servicemen were 62.6%, 21.1% and 43.2% respectively. Table 2 shows the demographics of the respondents. The majority of the respondents were males aged between 19-23 years old, reflecting the typical profile of our conscript military. General soldiers were of significantly lower education level and socio-economic status (as measured by the type of housing) compared to the other exposure groups.

**Table 2:** Demographics of study population

Demographics	Overall (%) (n = 1063)	Patients (%) (n = 47)	Contacts (%) (n = 244)	Healthcare Workers (%) (n = 331)	General Servicemen (%) (n = 441)	p-value*
**Mean Age (SE)**	21.4 (0.2)	20.6 (0.3)	20.6 (0.1)	23.2 (0.4)	20.5 (0.3)	< 0.001

-Inter quartile range	19 to 21	20 to 21	20 to 21	20 to 23	19 to 20	NA

-Range	17 to 61	17 to 27	18 to 30	18 to 61	17 to 57	NA

**Sex:**						

1. Male	1018 (95.8)	47 (100)	243 (99.6)	306 (92.4)	422 (95.7)	< 0.001

**Education:**						

1. Elementary/Middle School	530 (49.9)	18 (38.3)	96 (39.3)	103 (31.1)	313 (71.0)	< 0.001

2. High School	465 (43.7)	27 (57.4)	141 (57.8)	178 (53.8)	119 (27.0)	

3. Degree	67 (6.3)	2 (4.3)	6 (2.5)	50 (15.1)	9 (2.0)	

**Housing Type:**						

1. ≤ 3 room public flat	177 (16.7)	8 (17.0)	44 (18.0)	50 (15.1)	75 (17.0)	0.001

2. 4 room public flat	386 (36.3)	18 (38.3)	93 (38.1)	92 (27.8)	183 (41.5)	

3. 5 room public flat	309 (29.1)	13 (27.7)	65 (26.6)	105 (31.7)	126 (28.6)	

4. Private Property	167 (15.7)	7 (14.9)	38 (15.6)	74 (22.4)	48 (10.9)	

**Ethnicity:**						

1. Chinese	804 (75.6)	37 (78.5)	175 (71.5)	238 (71.9)	354 (80.3)	0.024

2. Malay	143 (13.5)	7 (14.9)	44 (18.0)	48 (14.5)	44 (10.0)	

3. Indian	62 (5.8)	2 (4.3)	9 (3.7)	28 (8.5)	23 (5.2)	

4. Others	31 (2.9)	0 (0.0)	9 (3.7)	11 (3.3)	11 (2.5)	

*Between patients, contacts, healthcare workers and general servicemen

### Scores

For the entire cohort, basic general knowledge of pandemic influenza A (H1N1-2009) was in general good with the exception of the low awareness by servicemen that influenza can be spread by touch (1.3%) and that it can present with nausea/vomiting (28.2%) or diarrhoea (20.7%). Knowledge regarding efficacy of mask use, oseltamivir and personal hygiene measures were good with more than 80% positive responses. Risk perception of illness was moderate, with almost half of the respondents believing they would be infected with pandemic influenza. For the practices, 32.8% of the cohort had used masks during the course of the pandemic (when ill or as prevention), 61.3% had previous seasonal influenza vaccination, 44.1% had practiced avoidance behaviors such as social distancing, and 62.2% practiced personal hygiene measures. Less than a third of respondents avoided seeking medical aid for influenza symptoms despite worries regarding picking up the illness at medical facilities.

Comparing between the 4 exposure groups of servicemen (Table 3), there was a significant difference between knowledge and practice scores. Close contacts had the highest knowledge score (71.7%), followed by healthcare workers (69.6%), patients (69.0%) and general servicemen (68.8%) (p = 0.004). Patients had the highest practice scores (58.8%) followed by healthcare workers (51.1%) and contacts (50.5%), while general servicemen had the lowest practice score (47.5%) (p < 0.001). There were no significant differences in attitude scores between the cohorts.

**Table 3:** Scores and zero-order correlation between scores for knowledge, attitudes and practices towards pandemic influenza A (H1N1)

	Overall % (SE)	Patients % (SE)	Contacts % (SE)	Healthcare Workers % (SE)	General Servicemen % (SE)	p-value*
**Scores**						

Knowledge Score	69.7 (0.5)	69.0 (2.5)	71.7 (1.7)	69.6 (1.5)	68.8 (1.5)	**0.004**

Attitudes Score	70.9 (0.4)	73.5 (2.6)	70.8 (1.8)	71.5 (1.6)	70.3 (1.5)	0.397

Practice Score	49.8 (0.5)	58.8 (2.9)	50.5 (2.0)	51.1 (1.8)	47.5 (1.8)	**< 0.001**

	**Overall**	**Patients**	**Contacts**	**Healthcare Workers**	**General Servicemen**	

**Correlation between scores**						

Knowledge Score with Attitudes Score	**0.21^†^**	0.31**	**0.19^†^**	**0.19^†^**	**0.22^†^**	NA

Attitudes Score with Practice Score	**0.12^†^**	0.23	0.00	**0.24^†^**	0.09	NA

Knowledge Score with Practice Score	**0.27^†^**	**0.46^†^**	**0.27^†^**	**0.28^†^**	**0.19^†^**	NA

*Between patients, contacts, healthcare workers and general servicemen

**Correlation is significant at the 0.05 level (2-tailed)

^†^Correlation is significant at the 0.01 level (2-tailed)

^††^Corrected for age, sex, education, housing and ethnicity

A greater proportion of patients used masks (68.5%) compared to the other groups (29.5-32%) (p < 0.001). Healthcare workers had the highest seasonal influenza vaccination uptake (81%) as compared to between 51-55% in other groups (p < 0.001). Contacts had the highest practice of avoidance behaviors (51.3%) as compared to 50.2%, 43.2% and 40.2% in patients, healthcare workers and general servicemen respectively (p < 0.001). General servicemen had the lowest mask usage (32.0%), vaccination uptake (51.0%) and practice of avoidance behaviors (40.2%).

### Predictors of Scores

From the univariate analyses, significant predictors for higher practice scores included female sex, exposure group (patients, contacts and healthcare workers compared to general individuals), ethnicity (Malay and Indian compared to Chinese), older age group, private housing compared to 3 room flats, and higher knowledge and attitude scores. The significant predictors of higher attitude scores were ethnicity (Malay compared to Chinese) and higher knowledge scores. The significant predictors for higher knowledge scores were contacts and healthcare workers, older age group, higher education levels, and private housing compared to 3 room flats.

From the multivariate analyses adjusting for potential confounders (Table 4), the final significant predictors of higher practice scores were higher knowledge scores (p < 0.001), Malay ethnicity (p < 0.001) and exposure group - patients (p < 0.001), contacts (p = 0.040) and healthcare workers (p = 0.010). Servicemen with higher education level (ie. University degree) had significantly lower practice scores (p = 0.042). The final significant predictors for higher attitudes scores were Malay ethnicity (p = 0.014) and higher knowledge scores (p < 0.001). The final significant predictor for higher knowledge score was being a contact (p = 0.007).

**Table 4:** Multivariate linear regression of scores for knowledge, attitudes and practice towards pandemic influenza A (H1N1)

		Practice Score			Attitudes Score			Knowledge Score		
		**β**	**95% CI for β**	**p-value**	**β**	**95% CI for β**	**p-value**	**β**	**95% CI for β**	**p-value**

**Sex**	Male	-2.27	-8.97 to 4.43	0.506	1.78	-4.18 to 7.73	0.558	2.42	-3.30 to 8.13	0.407

**Status**	Patients	**11.03**	**6.06 to 16.00**	**< 0.001**	3.30	-1.11 to 7.71	0.142	0.46	-3.77 to 4.69	0.831
	
	Contacts	**2.85**	**0.14 to 5.56**	**0.040**	0.588	-1.82 to 3.00	0.633	**3.19**	**0.88 to 5.50**	**0.007**
	
	Healthcare Workers	**3.39**	**0.78 to 6.00**	**0.011**	1.17	-1.14 to 3.48	0.323	0.79	-1.43 to 3.01	0.485
	
	General Servicemen*	NA			NA			NA		

**Ethnicity**	Others	-0.93	-7.02 to 5.14	0.762	3.08	-2.32 to 8.48	0.263	-3.63	-8.80 to 1.55	0.170
	
	Indian	2.33	-2.09 to 6.75	0.301	0.43	-3.49 to 4.36	0.829	1.46	-2.31 to 5.23	0.447
	
	Malay	**6.17**	**3.20 to 9.13**	**< 0.001**	**3.30**	**0.67 to 5.92**	**0.014**	-0.63	-3.15 to 1.89	0.626
	
	Chinese*	NA			NA			NA		

**Age**		0.25	-0.01 to 0.50	0.057	0.11	-0.11 to 0.34	0.330	0.22	-0.002 to 0.43	0.053

**Education**	High School	-0.03	-2.30 to 2.25	0.982	-1.50	-3.52 to 0.52	0.146	1.73	-2.31 to 5.23	0.080

	Degree	**-5.01**	**-9.84 to -0.18**	**0.042**	-3.82	-8.09 to 0.47	0.081	2.55	-3.15 to 1.89	0.223

	Elementary/Middle School *	NA			NA			NA		

**Housing**	4 room public flat	-1.08	-3.98 to 1.83	0.467	-1.57	-4.15 to 1.01	0.232	0.70	-1.78 to 3.17	0.582

	5 room public flat	-2.69	-5.75 to 0.37	0.084	-0.09	-2.80 to 2.63	0.951	0.50	-2.11 to 3.11	0.709

	Private Property	-2.41	-6.07 to 1.24	0.196	-0.85	-4.10 to 2.40	0.610	1.59	-1.53 to 4.71	0.316

	≤ 3 room public flat*	NA			NA			NA		

**Knowledge Score**		**0.30**	**0.22 to 0.37**	**< 0.001**	**0.21**	**0.14 to 0.28**	**< 0.001**			
			
**Attitudes Score**		0.04	-0.03 to 0.11	0.280						

*Reference group

### Correlation of Scores

The strongest overall correlation was between knowledge and practice scores (r = 0.27, p < 0.01), followed by knowledge and attitudes scores (r = 0.21, p < 0.01). The weakest correlation was between attitudes and practice scores (r = 0.12, p < 0.01) (Table 3). All 4 exposure groups had significant correlation between knowledge and practice scores, as well as knowledge and attitudes scores. Only healthcare workers had a significant correlation between attitudes and practice scores (r = 0.24, p < 0.01).

## Discussion

Our study provides evidence on the correlation between knowledge, attitudes, and practices among different exposure groups. This has substantial implications for public health educators and planners in implementing pandemic preparedness plans. It was evident that the knowledge score was the main predictor of the attitude and practice scores with strong correlation between knowledge and practice scores and knowledge and attitude scores. On the other hand, attitude scores alone did not predict practice score and the correlation between attitude and practice scores was weak. This shows that good knowledge is important to enable individuals to have better attitudes and practices in influenza risk reduction. In a previous study on SARS, better knowledge was also found to equate with better adoption of precautionary practices [6]. Clear communication and provision of updated information also helped improve vigilance and preparedness during the current pandemic [11]. A recent study found that educating the public about specific actions to reduce risks and communicating about the government's plans and resources helped to improve compliance to good practices [9]. Of interest, higher educational status in our cohort was a significant negative predictor of good practice, showing that educational status alone does not determine behaviours. Two previous studies on influenza [9] and SARS [2] also showed that education level did not have any effect on uptake of recommended behvioural patterns. Regarding influenza vaccine uptake and education level, some studies have showed that a higher education level resulted in higher influenza vaccination uptake [20,21], while another study on influenza vaccination uptake showed varying influence of education levels on influenza vaccination in different countries [22]. As such, it is important to focus on inculcating the correct knowledge to individuals as it will influence both attitudes and practices. On the other hand, positive attitudes on its own may not translate into desired behavioral change in the absence of adequate knowledge.

For the exposure groups, influenza cases had the highest practice scores of all 4 groups. Use of masks was also highest among the influenza cases. Having been infected with pandemic influenza appears to have a substantial impact in behavior and adopting risk reduction practices. Although most if not all of these influenza cases will not be re-infected by the same pandemic virus again, adopting these practices will place them and their close contacts at lower risk for other influenza and respiratory virus infections.

At the same time, healthcare workers and contacts of influenza cases also had higher practice scores compared to general servicemen. Vaccination uptake was highest among healthcare workers and avoidance behaviors were the highest among contacts of influenza cases. Healthcare workers and contacts have had greater and more direct exposure to influenza cases compared to the general population and this first-hand experience may have resulted in behavioral changes. This possibly reflects the effect of actual real-life experiences with influenza on individual behavior. It will therefore be important to determine solutions to instill the same level of positive behaviors in the general population without the need for prior infection or the personal experience such as being close contacts or healthcare workers. One possible solution would be the sharing and imparting of personal experiences to the general community

Ethnicity may also play a role in determining practices. We found that Malays (an ethnic minority) had significantly higher positive practices as compared to the Chinese (the ethnic majority). The Indians (another ethnic minority) also had higher practice scores as compared to the Chinese although this was not statistically significant. A previous study also reported that the ethnic minority groups had a 3.2 times higher likelihood of making recommended changes during this current influenza pandemic [9]. Another multi-ethnic study on a different subject (terrorism) showed that worry and voidance behaviours were more common among minority groups, suggesting that this effect may be due to shared perceptions of vulnerability or low levels of control [20] but further research is required to determine the actual causes for this phenomenon during epidemics. Overall efforts at increasing positive behaviours should therefore be rolled out to the entire population, with special focus on the ethnic majority.

We found that age, sex, and housing-type (as a proxy of socio-economic status) did not predict knowledge, attitudes or practices. However, the vast majority (> 80%) of our participants were from the 19-23 age group due to the inherent nature of the military and our study was not structured to detect any differences due to extremes of age. In another pandemic study in the United Kingdom, younger age was found to have greater uptake of recommended behaviors but not for sex and household income [9]. However, another study on behavioral changes during SARS found that the older age had an increased beneficial effect on behavioral change but not sex [2]. This shows the differences in behaviors in different settings and towards different threats. Future studies need to be performed in different age groups in specific settings to determine the actual platforms for intervention.

The possible lack of representativeness of a military cohort to the general population is an inherent limitation of this study, especially for the overall age structure. However, it does represent the behaviors of an important age group for the 2009 influenza pandemic, which affects mostly children and young adults. The questionnaire was also administered over a period of time and individuals' responses may have changed across time as they are exposed to different messages across time, include messages highlighting the pandemic's mild nature. We attempted to reduce this by starting the survey after the pandemic's peak and concluding it before the epidemic subsided. The response rate of 34.8% may also be of concern, but the study was sufficiently powered for all groups except influenza cases which numbers were smaller. Interestingly, the response rates among cases/contacts was high, and lower among healthcare workers, which may itself suggest behavioral differences which should be further studied. Given the anonymous nature of the survey, we were not able to obtain any data about non-responders. Finally, our study was a cross-sectional survey and may not have been able to assess the true association between knowledge, attitudes and practices, and future cohort studies should be considered to validate the findings.

## Conclusion

Knowledge is a significant influence on attitudes and practices in a pandemic, and personal experience influences practice behaviors. Efforts should be targeted at inculcating relevant knowledge and educating the general population to improve practices in the current pandemic, as well as for future epidemics.

## Competing interests

The authors declare that they have no competing interests.

## Authors' contributions

JY, VJL and PCT conceived the study, collected the data, performed the analysis, and wrote the manuscript together. TYY collected the data and participated in the manuscript writing. TPN performed the analysis and participated in the manuscript writing. All authors have read and approved the final manuscript and the manuscript is currently not submitted for publication elsewhere.

## Pre-publication history

The pre-publication history for this paper can be accessed here:

http://www.biomedcentral.com/1471-2458/10/442/prepub

## References

[B1] World Health Organisation Pandemic (H1N1) 2009 - update 81 2009 http://www.who.int/csr/don/2009_12_30/en/index.html

[B2] Tang CSK Wong CY An outbreak of the severe acute respiratory syndrome: predictors of health behaviours and effect of community prevention measures in Hong Kong, China Am J Public Health 2003 93 1887 8 10.2105/AJPH.93.11.1887 14600058 PMC1448068

[B3] Lau JTF Yang X Tsui H Kim JH Monitoring community responses to the SARS epidemic in Hong Kong: from day 10 to day 62 J Epidemiol Community Health 2003 57 864 70 10.1136/jech.57.11.864 14600111 PMC1732318

[B4] Tang CSK Wong CY Factors influencing the wearing of facemasks to prevent the severe acute respiratory syndrome among Chinese in Hong Kong Prev Med 2004 39 1193 10.1016/j.ypmed.2004.04.032 PMC7133369 15539054

[B5] Leung GM Ho LM Chan SK Ho SY Bacon-Shone J Choy RY Hedley AJ Lam TH Fielding R Longitudinal assessment of community psychobehavioral responses during and after the 2003 outbreak of severe acute respiratory syndrome in Hong Kong Clin Infect Dis 2005 40 1713 20 10.1086/429923 15909256

[B6] Leung GM Quah S Ho LM Ho SY Hedley AJ Lee HP Lam TH A tale of two cities: community psychobehavioral surveillance in Hong Kong and Singapore during the severe acute respiratory syndrome epidemic Infect Control Hosp Epidemiol 2004 25 1033 41 10.1086/502340 15636289

[B7] Lau JT Kim JH Tsui HY Griffiths S Anticipated and current preventive behaviors in response to an anticipated human-to-human H5N1 epidemic in the Hong Kong Chinese general population BMC Infect Dis 2007 18 10.1186/1471-2334-7-18 17359545 PMC1845150

[B8] Jones JH Salathe M Early assessment of anxiety and behavioural response to novel swine origin influenza A (H1N1) PLoSOne 2009 4 e8032 10.1371/journal.pone.0008032 PMC2779851 19997505

[B9] Rubin GJ Amlot R Page L Wessely S Public Perceptions, Anxiety, and Behaviour Change in Relation to the Swine Flu Outbreak: Cross Sectional Telephone Survey BMJ 2009 339 b2651 10.1136/bmj.b2651 19574308 PMC2714687

[B10] Seale H McLaws ML Heywood AE Ward KF Lowbridge CP Van D Gralton J MacIntyre CR The community's attitude towards swine flu and pandemic influenza Med J Aust 2009 191 5 267 9 19740048 10.5694/j.1326-5377.2009.tb02781.x

[B11] Lau JT Griffiths S Choi KC Tsui HY Widespread public misconception in the early phase of the H1N1 influenza epidemic J Infect 2009 59 122 7 10.1016/j.jinf.2009.06.004 19592114

[B12] Goodwin R Haque S Neto F Myers LB Initial psychological responses to Influenza A, H1N1 ("Swine flu") BMC Infect Dis 2009 9 166 10.1186/1471-2334-9-166 19807908 PMC2765446

[B13] Lee VJ Yap J Ong JB Chan KP Lin RT Chan SP Goh KT Leo YS Chen MI Influenza excess mortality from 1950-2000 in tropical Singapore PLoS One 2009 4 e8096 10.1371/journal.pone.0008096 19956611 PMC2779490

[B14] Macroepidemiology of Influenza Vaccination (MIV) Study Group The macro-epidemiology of influenza vaccination in 56 countries, 1997-2003 Vaccnie 2005 23 5133 43 10.1016/j.vaccine.2005.06.010 16039762

[B15] Ministry of Health, Singapore Weekly Infectious Disease Bulletin 2009 6 41 http://www.moh.gov.sg/mohcorp/uploadedFiles/Statistics/Infectious_Diseases_Bulletin/2009/2009_week_41.pdf

[B16] Goodwin R Haque S Neto F Myers LB Initial psychological responses to Influenza A, H1N1 ("Swine flu") BMC Infect Dis 2009 9 166 10.1186/1471-2334-9-166 19807908 PMC2765446

[B17] Rosenstock IM Strecher VJ Becker MH Social learning theory and the health belief model Health Educ Q 1988 15 175 183 3378902 10.1177/109019818801500203

[B18] Ajzen I Fishbein M Understanding Attitudes and Predicting Social Behavior 1980 Englewood Cliffs, NJ:Prentice-Hall

[B19] Singapore Department of Statistics Key indicators of resident households 2009 http://www.singstat.gov.sg/stats/themes/people/hhldindicators.pdf

[B20] Looijmans-van den Akker I van Delden JJ Verheij TJ van Essen GA van der Sande MA Hulscher ME Hak E Which determinants should be targeted to increase influenza vaccination uptake among health care workers in nursing homes? Vaccine 2009 27 4724 30 10.1016/j.vaccine.2009.05.013 19450642

[B21] Andrew MK McNeil S Merry H Rockwood K Rates of influenza vaccination in older adults and factors associated with vaccine use: a secondary analysis of the Canadian Study of Health and Aging BMC Public Health 2004 4 36 10.1186/1471-2458-4-36 15306030 PMC514709

[B22] Endrich MM Blank PR Szucs TD Influenza vaccination uptake and socioeconomic determinants in 11 European countries Vaccine 2009 27 4018 24 10.1016/j.vaccine.2009.04.029 19389442

[B23] Eisenman DP Glik D Ong M Zhou Q Tseng CH Long A Fielding J Asch S Terrorism-related fear and avoidance behavior in a multiethnic urban population Am J Public Health 2009 99 168 74 10.2105/AJPH.2007.124206 19008521 PMC2636595

